# Health on the Move (HOME) Study: Using a smartphone app to explore the health and wellbeing of migrants in the United Kingdom

**DOI:** 10.12688/wellcomeopenres.16348.1

**Published:** 2020-11-11

**Authors:** Robert W. Aldridge, Rachel Burns, Victoria Kirkby, Nadia Elsay, Elizabeth Murray, Olga Perski, Annalan M. Navaratnam, Elizabeth J. Williamson, Ramfis Nieto-Martínez, J. Jaime Miranda, Greg C. G. Hugenholtz

**Affiliations:** 1 UCL Public Health Data Science Research Group, Institute of Health Informatics, University College London, London, Camden, NW1 2DA, UK; 2 Primary Care & Population Health, Institute of Epidemiology & Health Care, University College London, London, NW3 2PF, UK; 3 Behavioural Science and Health, Faculty of Population Health Sciences, Institute of Epidemiology & Health Care, University College London, London, WC1E 6BT, UK; 4 Faculty of Epidemiology & Population Health, London School of Hygiene and Tropical Medicine, London, WC1E 7HT, UK; 5 LifeDoc Health, Memphis, TN, 38119, USA; 6 CRONICAS Center of Excellence in Chronic Diseases, Universidad Peruana Cayetano Heredia, Lima, Peru; 7 The George Institute for Global Health, UNSW, Sydney, Australia

**Keywords:** App, health, healthcare access, mhealth, migrant, migration, occupational health, refugee, smartphone application, wellbeing.

## Abstract

**Background/Aim**: We have a limited understanding of the broader determinants of health of international migrants and how these change over time since migration to the United Kingdom (UK). To address this knowledge gap, we aim to conduct a prospective cohort study with data acquisition via a smartphone application (app). In this pilot study, we aim to 1) determine the feasibility of the use of an app for data collection in international migrants, 2) optimise app engagement by quantifying the impact of specific design features on the completion rates of survey questionnaires and on study retention, 3) gather preliminary profile health status data, to begin to examine how risk factors for health are distributed among migrants.

**Methods**: We will recruit 275 participants through a social media campaign and through third sector organisations that work with or support migrants in the UK. Following consent and registration, data will be collected via surveys. To optimise app engagement and study retention, we will quantify the impact of specific design features (i.e. the frequency of survey requests, the time of day for app notifications, the frequency of notifications, and the wording of notifications) via micro-randomised process evaluations. The primary outcome for this study is survey completion rates with numerator as the number of surveys completed and denominator as the total number of available surveys. Secondary outcomes are study retention rates and ratings of interest after app usage.

**Ethics and dissemination**: We have obtained approval to use consented patient identifiable data from the University College London Ethics Committee. Improving engagement with the app and gathering preliminary health profile data will help us identify accessibility and usability issues and other barriers to app and study engagement prior to moving to a larger study.

## Introduction

**Figure 1.**
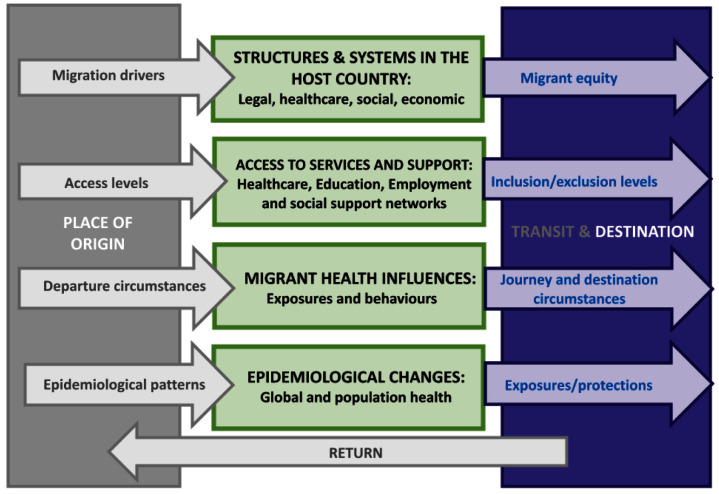
A conceptual framework for influences on migration and health adapted from UCL-Lancet Commission on Migration and Health ^1^. Boxes in green may apply at the individual, population or system level or a combination. The green boxes in the centre delineate four fundamental factors that influence the interactions between health and mobility. At the top, Structures and Systems, indicate the role of policies and legislation (e.g. immigration laws, human rights standards, entitlement schemes), the health system (e.g. design, funding, quality), socio-cultural factors (e.g. integration policies) and economic structures (e.g. employment access, financial protections). The second box, Access to Services and Support, refers to how migrants access and use health and non-health services and social services in their place of origin versus a destination location. Migrant Health Influences suggest the multiple exposures and behaviours that will influence migrants’ safety, health and wellbeing. The bottom box highlights Epidemiological Changes, expressing the rapid shifts that can occur at a population level, which may affect global, regional, national or specific population prevalence and incidence of communicable and non-communicable diseases and injuries. Grey arrows represent the migration-related health influences from the place of origin. The purple arrows represent migration or migrant-related health influences that emerge, decrease or increase at a transit stage and/or in the destination. Finally, the return arrow explicitly recognises the fact that many migrants will not remain in their destination country - the timing of which can vary considerably.

During their journey and after arrival in their host country, migrants experience large, rapid changes in the wider determinants influencing their health, including different legal, social, economic and health structures and systems; health service access and support; exposures and behaviours; and epidemiological changes associated with population mobility ( Figure 1) ^1^. In the UK there is a limited understanding of how these factors are distributed among migrants and how they change over time since migration. 

Gathering information about the health of migrants — which depends on the interaction between people, health systems and wider society — is challenging because migrant populations tend to be fluid in terms of mobility ^1^. Someone who has migrated recently may change address many times before settling. While many people migrate to the UK to rejoin their families, some migrants may initially not live in established households and may temporarily reside in short-term lets, hotels, hostels, shelters, caravan parks and other communal establishments.

One potential solution to improve data collection for research studies in mobile populations is to enable data collection via a smartphone application (app). Apps may have a particular use with mobile populations, such as migrants, with evidence suggesting that even the most excluded migrant populations have high levels (80–90%) of smartphone possession ^2^. During their migration journey, migrants use smartphones to communicate and access the internet, often using multiple sim cards, avatars and pseudonyms ^3^.

Smartphones are carried by migrants within the UK after arrival, thus they can inform us of their health needs and health determinants remotely — and continuously. An app that collects data can remove many barriers associated with conventional research studies, but we do not currently have good evidence on the acceptability of or engagement levels with research apps in migrant populations. Studies have been conducted in the general population that examine the barriers to and facilitators of engagement ^4^ and indicators of retention ^5^, with few studies specifically focused on the impact of survey content, schedules, and app notifications (frequency, timing and content) on engagement with mobile app research. Moreover, we have not found any studies conducted in migrant groups on engagement with research apps. We have designed an app for quantitative data collection in the UK migrant population, the Health On the MovE (HOME) app. Prior to running a large prospective longitudinal cohort study using the HOME app, we are conducting a pilot study — which this protocol describes — in order to test the feasibility of conducting research using an app in this study population and to fill the knowledge gap on how best to optimise engagement with app research in migrants.

## Aim and objectives

This study aims to pilot (or beta-test) the HOME app-enabled data collection platform to examine the health and wellbeing of migrants in the UK.

There are three main objectives of this pilot study:

1) To determine the feasibility of the use of the app for data collection in recent/long-term international migrants residing in the UK.
2) To optimise engagement in the study by quantifying the impact of specific app design features on completion of survey questionnaires and study retention.
3) To gather preliminary profile health status data, to begin to examine how risk factors for health and wellbeing are distributed among migrants and how they vary over time since their migration to the UK.

## Protocol

### Study design

This will be a longitudinal study with embedded micro-randomised process evaluations with each participant followed up for three months.

### Study recruitment

We will conduct a social media recruitment campaign working with partners in the UK that have an active online presence and work with migrants. An animated promotion video ( https://youtu.be/r314pjzxmn8) was made to maximise the recruitment potential and help explain the study and its aims. Participants may also be approached through third-sector organisations that work with or support migrants in the UK who we will work with to distribute information to potential participants — either via email or using printed study materials.

Once the study is launched, any eligible individual will be able to download the HOME app on Apple app or Google Play stores. Explicit consent will be obtained as part of the study registration process on the app.

### Eligibility criteria

To be eligible for the study, individuals need to be at least 18 years old, born outside of the United Kingdom and currently living in the UK. With this, we aim to reach migrants who are UK residents and are settled or are in the process of settling here.

For this study protocol, we define migrants as people born outside of the UK that are currently residing in the UK, regardless of the characteristics of migration (eg. when it occurred, at what point during their lifetime, and for how long), their reason to migrate and their migration status. This includes people who either have chosen to migrate (e.g. to work, study, or join families) or those who may have been forced to migrate due to conflict, persecution or environmental disasters (e.g. refugees and asylum seekers).

### Consent

Before consenting to participate, individuals are given in-app information on time commitment, benefits and disadvantages to participation, data collection, privacy and data protection, modes of dissemination of study findings and follow-up, and on how to contact the team or to withdraw from the study. Explicit consent is sought for the collection of data about physical, mental, and sexual health. 

### Registration

Directly following the consent process and registration, participants will be asked to complete a baseline demographics survey.

### Data collection

Personal data will be collected using the app, that will have been downloaded onto the participants’ smartphone. Personal data collected will include demographic information, such as name (first and surname), email, sex, date of birth, country of origin, ethnicity, and visa category. The participant will then be asked — once or twice weekly — to complete a short health status survey, lasting between 15 to 60 seconds (see *Extended data* File 1) ^6^. In addition to these short surveys, once a week, participants will be asked to fill in one of the 13 surveys that cover health, social, work or lifestyle-related topics (5-minute surveys; see *Extended data* File 1) ^6^. The topics of these surveys have been previously identified as knowledge gap areas by the UCL-Lancet Commission on Migration and Health and after discussions with migrants and relevant thematic experts following focus group sessions. All of the 13 surveys to be administered are validated surveys, or have taken questions from a validated survey and adapted them for the population and the app. Participation in the pilot study will last a total of 12 weeks. Migrants will be asked to complete weekly surveys via notifications from the HOME app.

### Participant engagement and retention

Micro-randomised process evaluations (MRPE) will be used to optimise participant engagement and retention in the final design of the study (see below). Structured feedback on engagement will be obtained through a short, interest-focused survey, administered once per week within the app. In a separate focus group study, we will seek feedback from migrants on barriers and enablers to engagement with the app. Participants who complete less than 20% of surveys during the three months of the study, will be followed up via email to ask about reasons for discontinuation to examine issues of acceptability.

Participant engagement and retention will be fostered by sending out communications via email and on our website every few weeks containing general findings throughout the course of the study. These regular communications were added as a direct result of feedback from focus groups — run to inform the design of the study — in which participants expressed the desire for regular updates about the study's findings. We will also continue to engage and promote the study throughout.

### MRPE

In order to increase survey completion rates and reduce selection bias (via a loss to follow up), we will conduct a series of micro-randomised process evaluations that are designed to address evidence gaps that we identified prior to starting the study ^4^ and after feedback from our focus group participants:

1. Schedule of short surveys — twice weekly (e.g. day 3 and 7) vs. weekly (e.g. day 7).
2. Timing of initial survey notification — which appears the next day if the survey has not yet been completed — 16.00 vs. 21.00
3. Timing of survey notification reminders: reminders to be sent to those not having completed a survey 1 day vs. 3 days after initial notification of the survey.
4. Text content of initial survey notifications: Two versions of notification text to be drafted — one using conventional wording and a second that is informed by Barriers to and Facilitators of Engagement With Remote Measurement Technology ^4^ and the COM-B model ^7^.
5. Text content of reminder survey notifications: Two versions of notification text to be drafted — one using conventional wording and a second that is informed by Barriers to and Facilitators of Engagement With Remote Measurement Technology ^4^ and the COM-B model ^7^.

**Figure 2.**
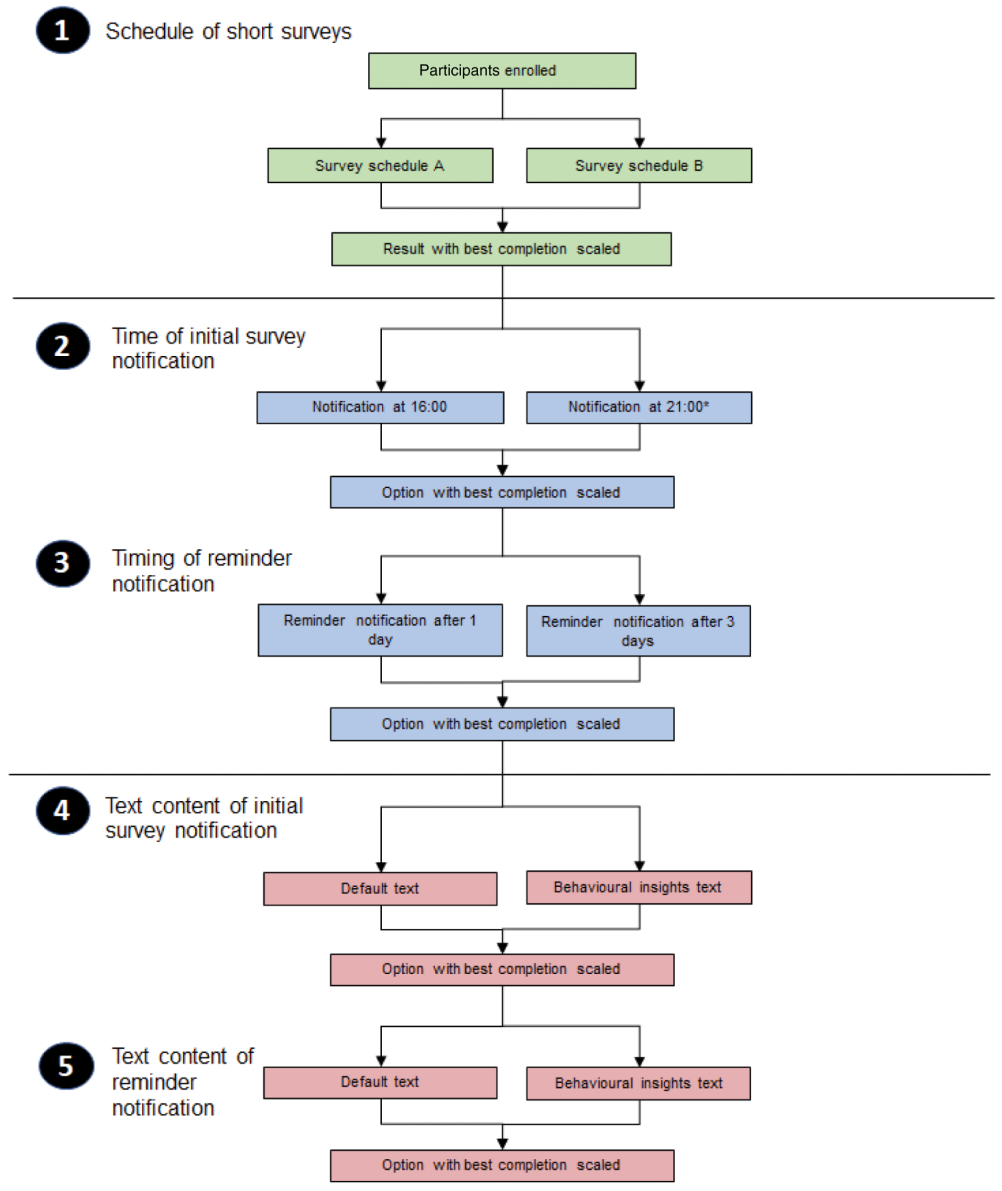
Flow diagram of study plan of micro-randomised process evaluation studies. *default schedule at baseline e.g. prior to running the test on. Survey schedule A is twice-weekly short surveys and schedule B is once-weekly short surveys.

Figure 2 outlines how micro-evaluations will be conducted sequentially and illustrates how evaluation 2 (Timing of survey notification reminders) will only begin once we have statistical power to answer evaluation 1 (Schedule of short surveys — twice-weekly vs. weekly) in terms of survey completion rates.

### Primary outcomes

The primary outcome for this study is survey completion rates with numerator as the number of surveys completed and denominator as the total number of available surveys.

### Secondary outcomes

1) Proportion of participants completing greater than 80% of surveys during the three months of the study.
2) Weekly ratings of “Interest” after app usage. The affective state of “Interest” has previously been identified as a significant predictor of participant engagement with mobile apps ^8^.

### Participant timeline

**Figure 3.**
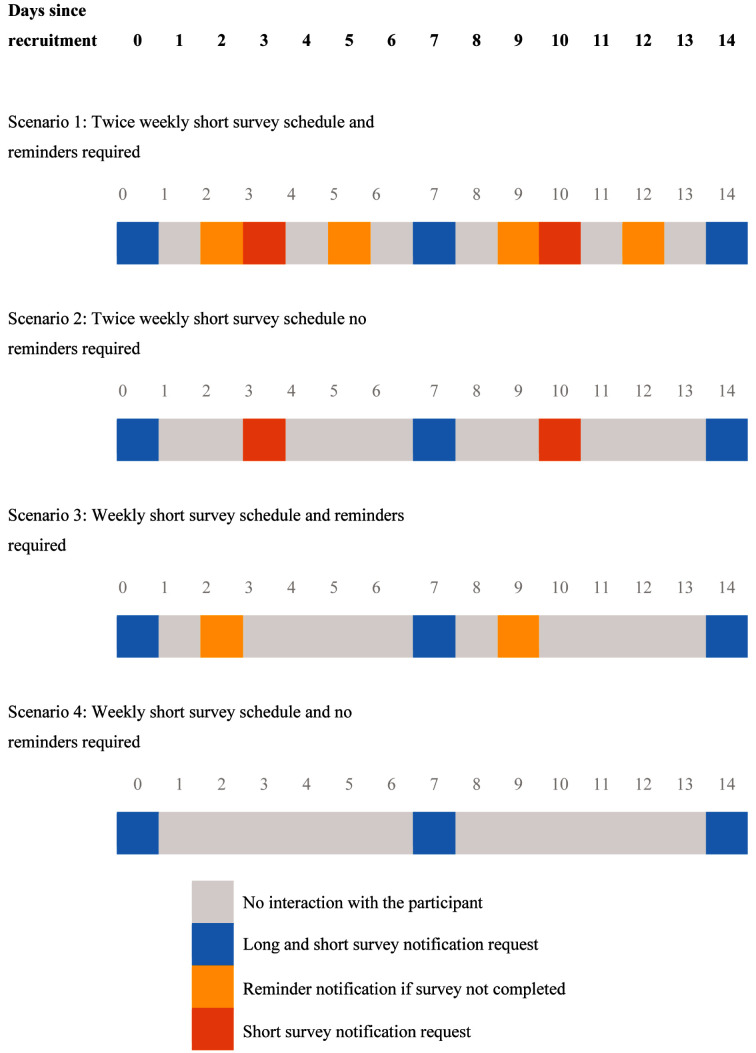
Participant timeline.

Figure 3 illustrates a series of typical participant timeline interactions with the HOME app timelines under different scenarios, including twice-weekly short survey schedules with reminders required and weekly short surveys with no reminders required.

### Assignment of interventions

***Allocation.*** Participants will be allocated to the different micro-evaluations using the A/B testing feature on Google Firebase which uses computer-generated random numbers. These allocations will occur during the recruitment and then again after the completion of each MRPE. Participants and the study analyst will be blinded to the allocation.

### Data management and analysis

***Data flow and management.*** Data flows and data management are described in detail in *Extended data* File 2 ^6^, including those for participant registration, survey notification, survey data collection, and data extraction. All data will be securely transferred by HTTPS and stored on secure servers through Amazon Web Services (AWS) and the UCL Data Safe Haven.

***Bias.*** To minimise measurement bias, we have used survey questionnaires previously validated wherever possible. The series of micro-randomised process evaluations we are undertaking aim to reduce bias in the main study by increasing engagement and reducing loss to follow up in the study. 

***Analysis plan.*** For the HOME app, there are five different aspects of the app which we wish to assess sequentially, in terms of their effects on response rates. Table 1 lists these five aspects, in the order planned for assessment. In each case, the response rate (baseline completion) is assumed to be 20%. For each modification, we have assumed an optimistic difference (the extent to which we hope the new version will increase response rates), and a pessimistic difference (the extent to which the new version may decrease response rates). For example, for the survey schedule, we expect 20% of migrants to complete the questionnaire using the standard version of the app. We hope that 40% of migrants who get more frequent notifications will complete the survey. However, if participants find the notifications intrusive it may be that only 5% of migrants will complete the questionnaire under the more frequent notifications condition.

**Table 1.** Parameters for power calculations for the migrant app.

	Assumed completion rates under the two options			Approximate probability of choosing better option under proposed design	
Test	Baseline completion	Optimistic difference	Pessimistic difference	Optimistic difference	Pessimistic difference
Survey schedule	20	20	5	90%	65%
Time of initial notification	20	20	5	90%	65%
Time of reminder notification	20	20	5	90%	65%
Text content of initial notification	20	10	5	75%	65%
Text content of reminder notification	20	10	5	75%	65%

Rather than power the study for traditional hypothesis tests for these 5 aspects relating to optimising the app, for each aspect we will apply a decision rule which chooses the option which leads to an expected loss in subsequent responses which is less than a pre-specified threshold of acceptable loss. This decision will be made as soon as sufficient information is available to make that decision. Additionally, a pre-specified maximum number of participants (per aspect) will be defined. Once this is reached, the option leading to the lowest expected loss (whether this is under the threshold or not) will be chosen.

Due to a lack of closed-form power calculations for this approach, the simulation was used to decide (i) the initial number of participants required prior to first calculating the expected loss under each option, (ii) the maximum participants per aspect. Using a threshold of 1% for an acceptable subsequent loss in responses, simulation studies showed that taking an initial group of 10 participants, updating the decision rule as data accumulates, and making a decision with a maximum of 50 participants per aspect, gives a high probability of choosing the correct option (analogous to the power) for each aspect ( Table 1). The lowest probability occurs if the completion rate is 20% under one option and 15% under the other, in which case the proposed design gives approximately 65% of choosing the better option. For slightly bigger effects (e.g. 20% completion under one option and 30% under the other), then the probability of choosing the better option is higher (75%).

In the first phase, we will summarise and compare baseline characteristics between the migrant group and the general population in the UK. We will review levels of missing data, but we anticipate that these will be analysed grouped as “not recorded”. We will analyse each MRPE after it has been delivered to 50 participants. 

## Ethics

We have obtained approval to use consented patient identifiable data from the University College London Research Ethics Committee (13571/003). We have conducted a Data Protection Impact Assessment (DPIA) and have registered our study with the UCL Data Protection Office — which has been reviewed and approved by the UCL Information Security Team. We will be obtaining individual consent from participants during their registration to the study.

## Dissemination

During the course of the study, we will send and publish newsletters — via email and on our website: www.homeappstudy.net — to the participants, presenting them with results of the study and other interesting facts about migrants in the UK.

## Discussion

The UCL HOME app study will prospectively gather information on socio-environmental determinants of wellbeing and health in the UK migrant population using an app. Cognisant of implementation challenges faced by digital health technologies ^9– 11^, we describe the design of a pilot study during which various ways to optimise acceptability, engagement and retention will be tested. Ensuring that digital tools meet user requirements, are accessible and easy to use, is important for future implementation — and are concerns which this study aims to address. Gathering preliminary socio-environmental and health profile data will help us understand whether there are issues around the generalisability of the pilot study. Results from the study will enable us to examine whether the pilot sample is representative of the larger UK migrant population, for example, by migrant status, country of origin, age and sex. The pilot study we describe has been designed to provide evidence that will inform the conduct of a larger HOME app study for the UK migrant population. Results from the pilot and subsequent larger cohort will enable us to begin to adapt the HOME app to study any migrant population worldwide under a structure that evaluates each step that will also help to guide the transcultural adaptation of the app.

The HOME app study design has several other advantages. To help ensure impact from the work, we have engaged with policymakers, national and international non-governmental organisations, migrants and the public throughout. To ensure impact from the work, we have engaged policymakers, national and international non-governmental organisations, migrants and the public throughout the design and conduct of the study. In designing this study, we held two workshops with six international migrants and 15 asylum seekers/refugees, to understand their views on the topics of interest, pressing needs, acceptability and appeal of the app, consent process, and dissemination of research data. Whilst our protocol has not been designed to fully capture many of the implementation science outcomes traditionally studied, it does strongly position the team towards a better understanding of the context and users, much needed for an adequate utilisation of implementation science frameworks ^11– 14^. We have involved eight national and international experts and policymakers working with migrant groups in the design and recruitment stages. We hope that this development work will enable us to create a long-term cohort that will help investigate inequities in socio-environmental determinants of health and wellbeing that matter to migrants both on a personal and population level.

Another advantage is the remote connection that the HOME app creates between the user and the researcher. Smartphones (and apps within it) are personal and always kept close, which creates convenience, facilitates honest disclosure and removes barriers to disclosing sensitive issues that can hamper the validity of research in often difficult to identify, mobile and hard-to-reach, unregistered or invisible populations. In this context, our team of researchers will be able to inform policymakers and civil society as to where and when inequalities are widening, so they can be targeted more rapidly with public and occupational health policies and programmes, community-level interventions or local improvements in policy and services.

There are several limitations to our study. A proportion of the migrant population will not have learned how to read or write or will not be fluent in English. This group may, therefore, face barriers to participating in the HOME app study in addition to having the worst health outcomes and the largest barriers to accessing healthcare in the NHS. We are not able to include data from individuals who are planning to leave or have left their countries, but who have not yet arrived in the UK, due to the complex practicalities of getting ethical approval from the governing bodies of respective countries where they currently reside. As a result, we will be unable to examine how socio-environmental determinants of health and wellbeing change as a direct result of the migration process (as conceptualised in Figure 1) at this stage of the study, but this remains our aim for the larger project. We also do not include data from non-migrant groups. As a result, the study findings will not be comparable to these groups, a comparison which might have helped differentiate between the effects of migration versus ethnicity on barriers to access to services, stigma and discrimination. Our proposed study will not be able to link changes in socio-environmental determinants of health to healthcare and mortality outcomes, but it will continuously measure whether participants experience changes in their mental and physical health, whether they have been injured (including occupational injuries) and the pathways used to access healthcare or health support services and how easy access has been. Ultimately, we hope to link this health information to other HOME app survey results in order to determine how social-environmental determinants might influence migrant health. Specific healthcare outcomes and mortality will be examined separately in the Million Migrants study ^15^ and in a population-based cohort study using Clinical Practice Research Datalink ^16^ (both studies are study part of RWA’s Wellcome Trust Fellowship, Public health data science to investigate and improve migrant health in the UK), but these studies will not be able to assess whether healthcare and mortality outcomes are affected by frequency of travel abroad, health service usage abroad, uncertainty in UK residence status, movement in the UK outside of England to access healthcare, or wider socio-environmental determinants of health. The UCL HOME app study will examine these factors. Data generated from the UCL HOME app study, the Million Migrant study and the analysis of the Clinical Practice Research Datalink will, therefore, complement each other and provide high-quality evidence on hospital-based events and mortality and the socio-environmental determinants driving them, which — when combined — will provide us with new, detailed insights into the health of this vital community in the UK.

## Data availability

### Underlying data

No underlying data are associated with this article.

### Extended data

UCL Research Data Repository: Extended data files for Health on the Move (HOME) Study - Using a smartphone app to explore the health and wellbeing of migrants in the United Kingdom. https://doi.org/10.5522/04/13049702 ^6^.

This project contains the following extended data:

- Extended data file 1 - surveys.pdf. (HOME App surveys.)
- Extended data file 2 - data flows.pdf. (HOME App data flow diagram.)

Extended data are available under the terms of the Creative Commons Attribution 4.0 International license (CC-BY 4.0).
