# Supplementary material for: Health on the Move (HOME) Study: Using a smartphone app to explore the health and wellbeing of migrants in the United Kingdom
Source: Wellcome Open Res. 2020 Nov 11;5:268. [Version 1] doi: 10.12688/wellcomeopenres.16348.1 (PMC8008349; doi:10.12688/wellcomeopenres.16348.1)
Supplement: Peer review history

# Peer review: Reviewer response for version 1

**DOI:** 10.21956/wellcomeopenres.17975.r42669

**Published:** 2021-03-26

**Authors:** Laura Goodwin

**Affiliations:** 1 Department of Psychological Sciences, University of Liverpool, Liverpool, UK

1. Really important research on an understudied population with the potential for high impacts on policy and practice from the research findings.
2. I was convinced by the argument that an app may be a suitable tool for data collection and I think most migrants will have a smartphone. One issue to consider is whether people would always have the credit for the data if they are on pay as you go contracts and is there an option to reimburse people for the data used to participate?
3. I had a few queries about the methodological approach and the difference between using the app as a suitable way to administer repeated measure of the same outcome (in line with the cohort design that is discussed) compared to the in-depth questionnaire data collections that will take place weekly. I wondered if these longer questions could put people off taking part in the repeated data collections of the key health data?
4. Regarding the questionnaires then I wasn't sure if all of them would be appropriate for the population and there are a lot of questions with some duplication across surveys. For example, there did seem to be some replication between the broader occupational health questions and those from the Labour Force Survey.
5. I also wasn't sure if the PHQ and other mental health measures have been validated to be culturally appropriate across different ethnic groups as representation of mental health symptoms has been established to vary across cultures.
6. The ethnicity measure also seems appropriate for a UK population more broadly, but may lack specificity in understanding the broader list of ethnic group that could be relevant for different migrant populations.
7. Finally, the MRPEs seem a good idea to understand which factors may impact on participation but from the explanation I wasn't completely clear on the design. Will each of these be conducted as a standalone comparison (not taking into account the previous condition someone was in, which could influence future participation) or will participants be allocated to a specific combination of the 5 conditions?
8. I hope that most of these issues should be fairly easy to resolve and I think this is a really valuable study. Additionally, the learning around encouraging recruitment will be relevant to a range of research areas.

Is the study design appropriate for the research question?

Yes

Is the rationale for, and objectives of, the study clearly described?

Yes

Are sufficient details of the methods provided to allow replication by others?

Yes

Are the datasets clearly presented in a useable and accessible format?

Not applicable

Reviewer Expertise:

Psychiatric Epidemiology.

I confirm that I have read this submission and believe that I have an appropriate level of expertise to confirm that it is of an acceptable scientific standard.

# Peer review: Reviewer response for version 1

**DOI:** 10.21956/wellcomeopenres.17975.r41799

**Published:** 2021-02-18

**Authors:** Qiaobing Wu

**Affiliations:** 1 Department of Applied Social Sciences, The Hong Kong Polytechnic University, Hong Kong, Hong Kong

This paper introduced an interesting study, Health on the Move (HOME) study, which employed an innovative methodological approach applying digital technologies in data collection. The authors provided clear and detailed description of the procedure of using a smartphone app to collect health and wellbeing data of migrants in the United Kingdom, a mobile population that was hard to track via traditional ways of data collection. Particularly, the study design included a micro-randomized process evaluation (MRPE) procedure, an embedded meaningful step that would optimize the design and operation of the app to maximize the response and retention rate of participants. Indeed, the study could have made a significant contribution to the field of migrant research with its innovative and well thought through methodological design.

 Generally, the study aims and objectives were clearly presented. The study design was appropriate to answer the research questions, and sufficient details of the method was described to allow easy replication. Although the paper stays with an introduction of the study and a description of the method to be used without providing any results of the study, it carries special scientific merit and can make a unique contribution to the research community considering that it is meant to be published as a study protocol. It does invoke the curiosity and interest of readers to expect reading further the findings of the study once it has been implemented, such as what kind of combination of survey and notification design could best reach and retain participants. I believe this study can be really informative for the design of future research by answering its research questions and achieving the list of study objectives.

Is the study design appropriate for the research question?

Yes

Is the rationale for, and objectives of, the study clearly described?

Yes

Are sufficient details of the methods provided to allow replication by others?

Yes

Are the datasets clearly presented in a useable and accessible format?

No

Reviewer Expertise:

Migration; health; social policy.

I confirm that I have read this submission and believe that I have an appropriate level of expertise to confirm that it is of an acceptable scientific standard.
